# A Typical Case of Atypical Disease: "Three Noes" Infective Endocarditis

**DOI:** 10.7759/cureus.65325

**Published:** 2024-07-25

**Authors:** Masashi Yokose, Takanobu Hirosawa, Keita Tsunashima, Taro Shimizu

**Affiliations:** 1 Department of Diagnostic and Generalist Medicine, Dokkyo Medical University, Mibu, JPN; 2 Department of Emergency and General Medicine, Dokkyo Medical University Nikko Medical Center, Nikko, JPN

**Keywords:** diagnostic error, typicality, three noes infective endocarditis, infective endocarditis, atopic dermatitis

## Abstract

Recognizing typical presentations of atypical cases is essential to reduce diagnostic errors and achieve better diagnoses. To better understand this, we report a typical case of “three noes” infective endocarditis (no left-sided, no intravenous drug use, and no intracardiac devices) with some different characteristics from well-known infective endocarditis. A 16-year-old boy with a history of atopic dermatitis presented with a one-month history of intermittent fever and shaking chills. The patient became afebrile with oral antibiotics, and initial investigations did not detect any evidence of bacterial infection, including heart murmurs and peripheral embolic manifestations. However, his symptoms relapsed one week after the cessation of antibiotics. A repeated workup revealed tricuspid valve infective endocarditis due to methicillin-susceptible *Staphylococcus aureus*. The relationship between atopic dermatitis and infective dermatitis has been reported in some literature, and clinicians should consider three noes infective endocarditis in patients with atopic dermatitis presenting with fever and unremarkable physical examination.

## Introduction

Infective endocarditis (IE) is one of the most severe infectious diseases with high morbidity and mortality [1]. While IE typically involves the left side of the heart, and there has been extensive data on left-sided IE, right-sided IE (RSIE) remains less understood [2]. Among cases of RSIE, some lack typical risk factors such as intravenous drug use (IVDU) or intracardiac devices, and they are known as three noes IE [3]. Clinical manifestations of three noes IE are nonspecific compared to left-sided IE; therefore, the diagnosis is often challenging [4]. We report a typical case of three noes IE in a patient with atopic dermatitis.

## Case presentation

A 16-year-old high school student was referred to the university hospital with a one-month history of intermittent fever and shaking chills. Three days before the initial visit, the primary care physician prescribed cefdinir for five days. Since then, the patient has been afebrile. The patient had atopic dermatitis and a history of childhood asthma without intracardiac devices. His family history was unremarkable. The patient did not smoke tobacco, drink alcohol, or use illicit drugs. There were no known allergies. His medications included montelukast, olopatadine, topical betamethasone, topical hydrocortisone, and heparinoid cream (a commonly used skin moisturizer in Japan).

On physical examination, the patient appeared well, with normal vital signs. There were no conjunctival petechiae or dental caries. The heart was normal. His skin revealed patchy scaly erythema on the scalp. The remainder of the examination was normal. Laboratory tests showed serum C-reactive protein level of 1.69 mg/dL (reference range ≤0.14 mg/dL), while the complete blood count and basic metabolic panel were unremarkable. The chest radiograph, abdominal ultrasound, and transthoracic echocardiography (TTE) were normal. Blood specimens were obtained for culture. Due to the lack of evidence of bacterial infections at this time, the patient was advised to stop cefdinir and make a follow-up appointment in one week.

**Figure 1:**
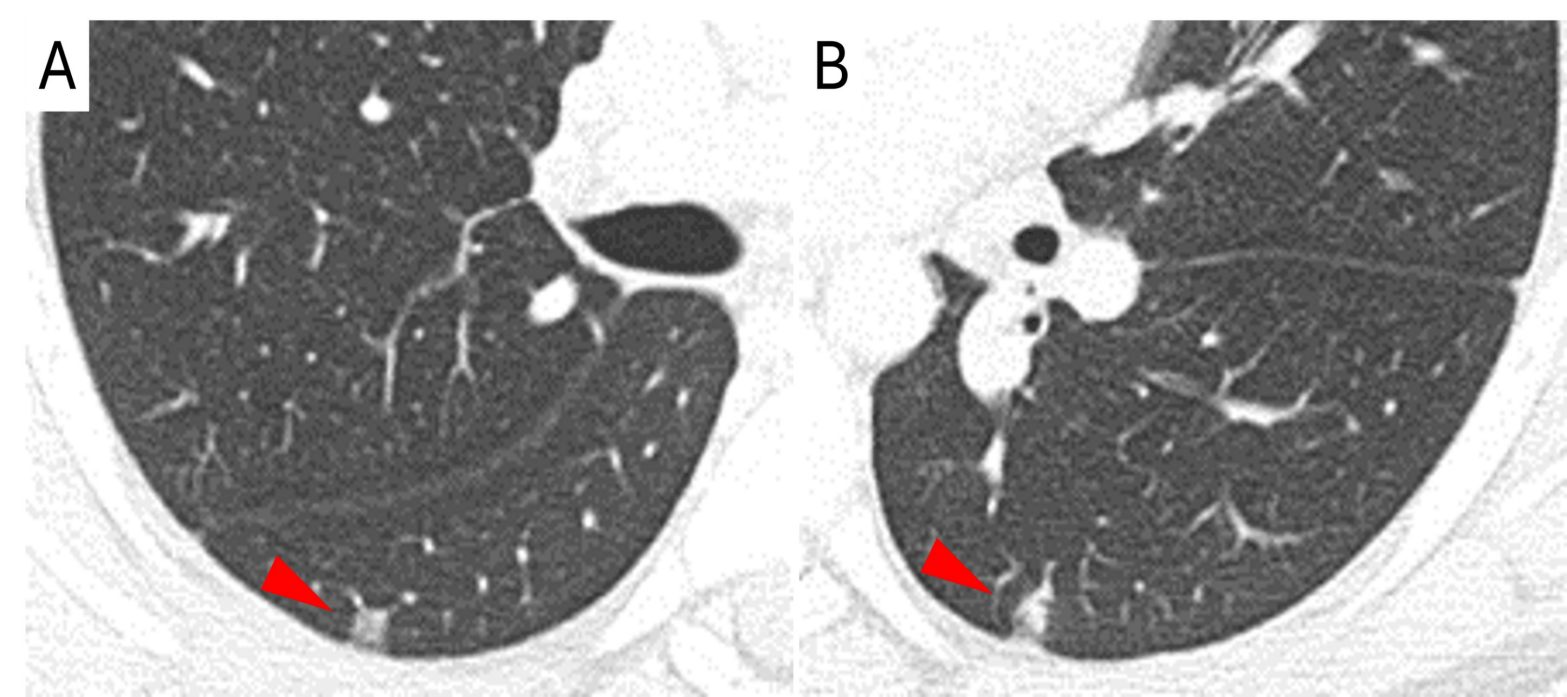
CT of the chest without contrast. CT of the chest without contrast on the day of admission revealed two wedge-shaped small nodules (arrowheads) in both right (A) and left (B) lung bases. CT: computed tomography.

Six days after the initial visit, which was one day before an appointment, the patient presented to the hospital due to a relapse of fever and shaking chills. The patient was exhausted but alert and oriented. His body temperature was 39.0°C, pulse 125 beats per minute, blood pressure 105/64 mmHg, respiratory rate 20 breaths per minute, and oxygen saturation 97% while breathing ambient air. A repeated physical examination revealed no new findings. The complete blood count showed a white blood cell count of 11,300/µL, hemoglobin level of 11.2 g/dL, mean corpuscular volume of 84.9 fL, and a platelet count of 173,000/µL. The C-reactive protein level was 10.5 mg/dL. The electrolyte levels were normal, as were the results of kidney function and liver function tests. Computed tomography (CT) of the chest without contrast revealed two wedge-shaped small nodules in the bilateral lung bases (Figure 1).

**Figure 2:**
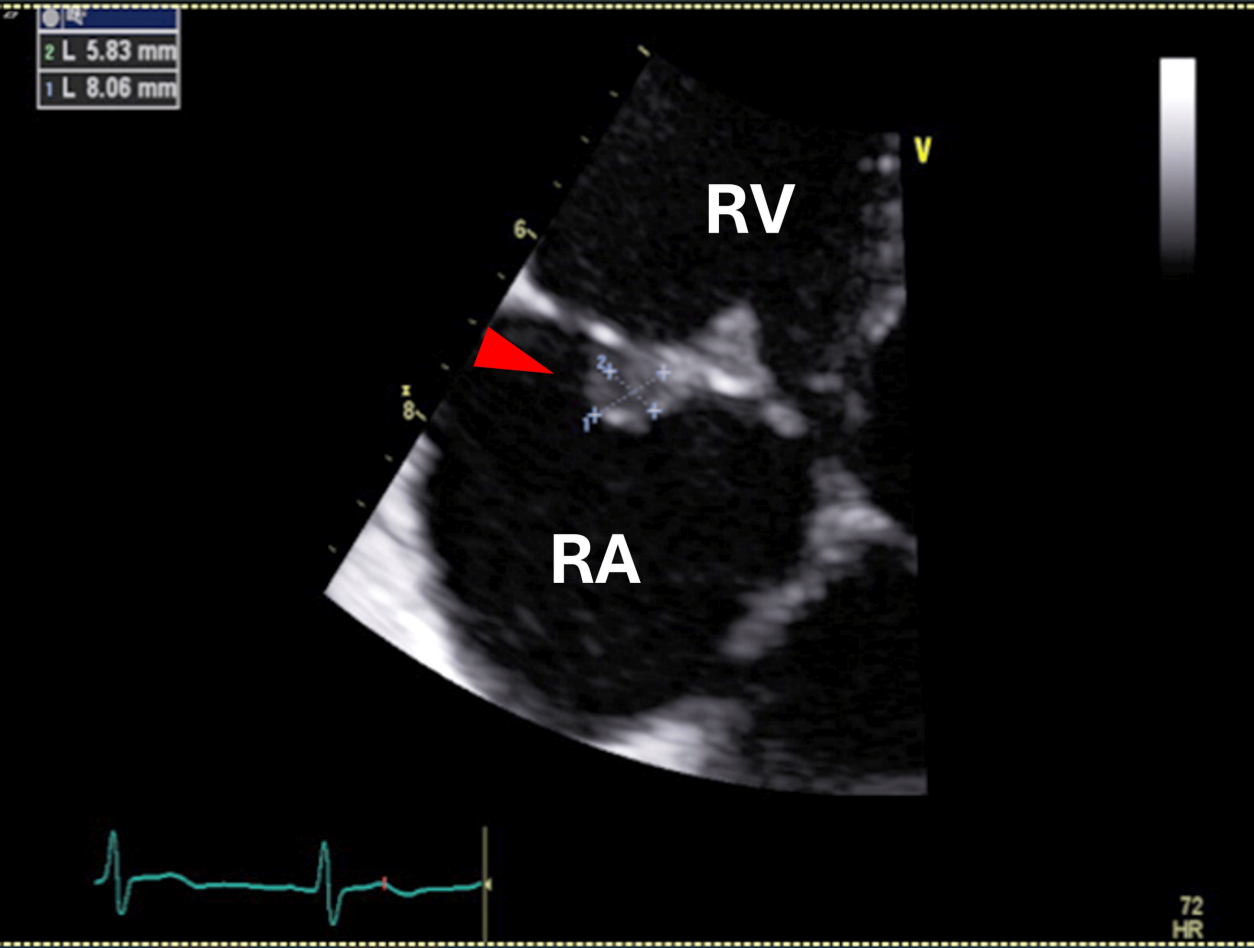
TTE on the day of admission. Repeated TTE on the day of admission showed an 8.0 X 5.8 mm mobile vegetation (arrowhead) on the tricuspid valve. RA: right atrium; RV: right ventricle; TTE: transthoracic echocardiography.

Contrast-enhanced CT of the abdomen showed normal results. Repeated TTE showed an 8.0 x 5.8 mm mobile vegetation on the tricuspid valve (Figure 2) with no vegetation on the left side of the heart.

Empirical treatment with intravenous ceftriaxone and vancomycin was started. While blood cultures obtained at the initial visit were negative, repeated blood cultures at admission grew methicillin-susceptible *Staphylococcus aureus* (MSSA). The patient was diagnosed with tricuspid valve IE due to MSSA and admitted to internal medicine wards. We switched antibiotics to intravenous cefazolin, and the patient underwent six-week intravenous antibiotics from the first day of negative blood culture. His primary care physician increased skin moisturizer for atopic dermatitis to prevent scratching. The patient returned to usual school activities, and follow-up TTE in two months showed no vegetation.

## Discussion

Isolated RSIE is an infection affecting the right heart chambers and valves, especially the tricuspid valve, without left heart involvement [2,4]. RSIE accounts for about 10% of all IE cases [2]. Most RSIE cases are associated with IVDU or intravenous lines and wires (e.g., pacemakers and implantable cardioverter defibrillators) [2-4]. A few patients with RSIE do not have these typical risk factors, and this atypical population is called the “three noes” IE group (no left-sided IE, no IVDU, and no intracardiac devices) [3]. This group is more likely to have some comorbidities such as renal failure or dialysis, diabetes mellitus, cancer, and chronic obstructive pulmonary disease [3]. *Staphylococcus aureus *and coagulase-negative staphylococci are the leading causes of RSIE [2-4]. Compared to left-sided IE, RSIE has some unique clinical manifestations. Patients with RSIE often show pulmonary manifestations such as chest pain, dyspnea, cough, hemoptysis, and septic pulmonary emboli (typically, peripheral wedge-shaped opacities on chest CT, as shown in this case) [3-7]. Pathologic murmurs are rare [5,6], and only 10% of patients show peripheral embolic manifestations [3,5]. Due to this atypical presentation, diagnosis is often delayed [4].

Atopic dermatitis is a common chronic eczematous skin disease affecting approximately 10% of the general population [8]. Due to multifactorial mechanisms, including an impaired skin barrier and abnormal cutaneous immune response, atopic dermatitis is relevant to cutaneous and extracutaneous infections [8]. A previous study from Japan showed that the prevalence of atopic dermatitis in patients with IE was 6.7% [9], and another study from the United States reported that atopic dermatitis was one of the potential risks of IE [8]. While older patients are more likely to have IE, IE associated with atopic dermatitis occurs in younger patients (median age: 27.5 years) [9,10]. *Staphylococcus aureus* is responsible for almost all cases due to colonizing patients’ skin [9].

In this case, typical characteristics of “well-known” IE were seemingly lacking: the patient was young and had no heart murmurs or peripheral embolic manifestations. In hindsight, however, the presentation was typical of three noes IE, and atopic dermatitis might be a potential risk factor. In addition, the preceding antibiotic treatment led to the negative result of the initial blood cultures, which complicated this case. Previous studies have shown that atypical presentations of any disease are associated with diagnostic errors [11]; therefore, clinicians should recognize patterns of “typical presentations of atypical cases” to reduce the burden of atypicality and achieve better diagnoses. When seeing patients with atopic dermatitis presenting subacute or chronic fever and an unremarkable physical examination, typical three noes IE might be the final diagnosis; thus, we should perform repeated TTE and obtain blood cultures without preceding antibiotic use.

## Conclusions

IE should be considered in patients with atopic dermatitis presenting subacute or chronic fever, even if they seem unremarkable on physical examination.
